# Purification, characterization, and anticoagulant mechanism of an anticoagulant protein from marine *Bacillus velezensis* B01

**DOI:** 10.3389/fmicb.2026.1857346

**Published:** 2026-05-26

**Authors:** Jingran Yu, Mingdi Yang, Haoyi Xu, Yankai Qin, Xuezheng Zhang, Xuguang Ruan, Fuyao Tong, Xiaoyan Cao

**Affiliations:** 1 College of Agriculture and Forestry, Linyi University, Linyi, Shandong, China; 2 Agricultural and Financial Service Center of Zaoyuan Town, Linyi, Shandong, China; 3 College of Medicine, Linyi University, Linyi, Shandong, China

**Keywords:** anticoagulant, anticoagulant protein, *Bacillus velezensis*, marine microorganism, thromboembolism

## Abstract

Anticoagulants are the primary drugs for the prevention and treatment of thromboembolism. However, most currently available anticoagulants have certain limitations, and there is an urgent need for novel anticoagulants. Marine microorganisms represent a valuable reservoir for the discovery of novel drugs. A marine microorganism capable of producing an anticoagulant protein was isolated from the mangrove mudflat sediment in Beihai City, China. The strain was classified and identified based on 16S rRNA gene sequencing and sequence alignment, combined with phenotypic characteristics. The anticoagulant protein was isolated and purified by ammonium sulfate precipitation, DEAE Sepharose Fast Flow anion exchange chromatography, and Sepharose CL-6B gel filtration chromatography. Its molecular weight was determined by SDS-PAGE (Sodium Dodecyl Sulfate-Polyacrylamide Gel Electrophoresis) and COSMOSIL 5Diol-120-II gel filtration chromatography. Physicochemical properties, hemolytic safety, and the effect on erythrocyte aggregation were also examined. To evaluate its anticoagulant effect, *in vitro* experiments including inhibition of fibrin formation and fibrinogen degradation were performed. The results showed that the strain was identified as *Bacillus velezensis* and designated as B01. The purified anticoagulant protein had a molecular weight of 27.2 kDa. Its optimal reaction temperature was 50 °C, and it exhibited good temperature stability in the range of 25–37 °C. The optimal pH was 9.0, and it remained stable under alkaline conditions, particularly at pH 9.0–10.0. Cu^2+^ and Fe^3+^ significantly inhibited the protein’s activity, whereas Zn^2+^, Ca^2+^, and Mn^2+^ promoted its anticoagulant activity. The protein showed no hemolytic activity and did not induce erythrocyte aggregation. It degraded fibrinogen with a chain degradation order of γ > Bβ > Aα, a pattern that is relatively rare. This anticoagulant protein has the potential to become a novel anticoagulant agent.

## 1 Introduction

Thromboembolic disorders have emerged as a leading cause of mortality and disability worldwide. Hemorrhagic thrombosis is recognized as the underlying etiology for one quarter of all global deaths (Raskob et al., 2014) and constitutes the primary pathogenic mechanism responsible for the majority of myocardial infarctions, strokes, and venous thromboembolisms (VTEs) (Wendelboe and Weitz, 2024). Currently, the clinical prevention and management of thrombotic diseases rely predominantly on anticoagulants, antiplatelet agents, and thrombolytic drugs. Despite their essential role in clinical practice, these agents are frequently associated with several limitations, including an elevated risk of bleeding, a narrow therapeutic window, the need for frequent coagulation monitoring, high costs, and pronounced antigenicity (Mackman, 2008; Weitz et al., 2012). Therefore, the discovery and development of novel anticoagulant and thrombolytic agents derived from natural resources, which offer high efficacy, low toxicity, and favorable safety profiles, hold important clinical value and practical importance.

In recent years, microbial-derived proteases have emerged as a promising direction in the research and development of anticoagulant and thrombolytic drugs, owing to their diverse sources, low production costs, and potential for oral administration (Gowthami and Jaya Madhuri, 2021). Chen et al. systematically reviewed the research progress on nattokinase and pointed out that fibrinolytic enzymes derived from microorganisms hold great promise for the development of functional foods and drugs, particularly in the field of cardiovascular diseases (Chen et al., 2018). Moreover, an increasing number of microbial fibrinolytic enzymes originating from marine and wetland environments have been reported. For instance, Zibin Ma et al. isolated the fibrinolytic enzyme BSFE1 from marine bacterium *Bacillus* sp. isolated from the South China Sea (Ma et al., 2024), while Zhao et al. identified a bifunctional enzyme possessing both anticoagulant and thrombolytic activities from marine fungi (Zhao et al., 2022). These findings suggest that microorganisms derived from marine and wetland habitats represent an important reservoir for novel anticoagulant proteases.

*Bacillus velezensis* has attracted considerable attention due to its robust capacity for synthesizing diverse secondary metabolites. Lu et al. purified and systematically characterized a fibrinolytic enzyme from *Bacillus velezensis* SN-14 (Lu et al., 2021). Zhou et al. isolated Velefibrinase from the marine-derived *Bacillus velezensis* Z01, which demonstrated potent thrombolytic effects both *in vitro* and *in vivo*, with no hemolytic activity (Zhou et al., 2022). However, those studies primarily employed fibrinolytic activity (i.e., the ability to degrade preformed fibrin clots) as the core evaluation metric, and the strains used were predominantly isolated from fermented foods or marine waters. To date, no reports have been published on the screening of *Bacillus velezensis* from the mangrove forests of the South China Sea, nor on the systematic investigation of the anticoagulant activity of its protease.

Mangrove ecosystems are unique wetland communities located in the intertidal zones of tropical and subtropical regions. The extreme environmental conditions in these ecosystems, such as high salinity and hypoxia, endow the microorganisms inhabiting the sediments with distinct metabolic diversity. In a systematic screening study, Fei et al. isolated 18 strains with antithrombotic activity from 125 culturable bacteria collected from mangrove plants on the mudflat of Beihai City, China, yielding a positive rate of 24.32% (Fei et al., 2020). This finding suggests that mangrove ecosystems harbor abundant microbial resources capable of producing anticoagulant active substances. In recent years, researchers abroad have also successfully isolated fibrinolytic enzyme-producing *Bacillus* strains from the soil of the Coringa mangrove forest in India, further confirming the potential of mangroves as a novel reservoir of fibrinolytic enzyme-producing microorganisms (Bhavana and Alok, 2024; Sompalli and Malaviya, 2024).

Kattula et al. systematically reviewed the mechanisms by which fibrinogen and fibrin contribute to hemostasis and thrombosis formation. Fibrinogen, the core substrate of the coagulation cascade, is composed of three pairs of polypeptide chains: Aα, Bβ, and γ. Upon thrombin cleavage, fibrinogen is converted into fibrin monomers, which then polymerize to form the thrombus scaffold. In this process, cross-linking of the γ chain plays a critical role in clot stability. Therefore, proteases that selectively degrade specific fibrinogen peptide chains can interfere with normal fibrin polymerization and exert anticoagulant effects (Kattula et al., 2017). Recently, Jiang et al. isolated a fibrinolytic enzyme, FEB-1, from *Bacillus amyloliquefaciens* that preferentially degrades the Aα and Bβ chains (Jiang et al., 2024). Gudzenko et al. reported that a protease from *Bacillus atrophaeus* preferentially degrades the Aα chain, with negligible activity toward the Bβ chain (Gudzenko et al., 2024). These differences in chain selectivity patterns further underscore the diversity of fibrinogen-degrading enzymes of microbial origin. Fibrinolytic enzymes reported from *Bacillus velezensis* mainly degrade the Aα and Bβ chains (Lu et al., 2021; Zhou et al., 2022). However, no protease from *Bacillus velezensis* with selective activity toward the γ chain has been reported to date.

In this study, a *Bacillus velezensis* strain secreting an anticoagulant protein was isolated from the sediment of mangrove forests in Beihai, China. Following purification, the physicochemical properties, biological safety, and anticoagulant activity of the protein were systematically investigated. In addition, its selective degradation characteristics toward the distinct peptide chains of fibrinogen were analyzed. This study aims to provide a scientific basis for the application of mangrove microbial resources in the development of anticoagulant drugs.

## 2 Materials and methods

### 2.1 Chemicals and reagents

Fibrinogen (from bovine plasma), phosphorylase b, ovalbumin, α-Chymotrypsinogen A, cytochrome C, and bovine serum albumin (BSA) were purchased from Sigma-Aldrich (St. Louis, MO, United States). Lyophilized thrombin powder was purchased from Zhejiang Hacon Pharmaceutical Co. Ltd. (Zhejiang, China). Heparin sodium was purchased from Solarbio Life Science (Beijing, China). All other chemicals were of analytical or sequencing grade.

Solution A: Glycine-NaOH buffer (0.02 M, pH 10).

Solution B: Glycine-NaOH buffer (0.02 M, pH 10) supplemented with 0.5 M NaCl.

Solution A1: 0.02 M disodium hydrogen phosphate-sodium dihydrogen phosphate buffer (pH 7.5).

Solution B1: 0.02 M disodium hydrogen phosphate-sodium dihydrogen phosphate buffer (pH 7.5). supplemented with 0.2 M NaCl.

All buffers were prepared with deionized water and the pH was adjusted at 25 °C using a calibrated pH meter.

### 2.2 Microorganism and cultivation conditions

A bacterial strain capable of producing anticoagulant protein was isolated from mangrove sediment along the coast of Beihai City, Guangxi, China. It was stored at −80 °C in 30% glycerol for preservation.

Seed medium (g/L): glucose 5, yeast extract 5, peptone 10, NaCl 5, pH 7.0–7.2. Erlenmeyer flasks (250 mL) containing seed medium (50 mL) were incubated at 37 °C and 160 rpm with an inoculum size of 4% (v/v).

Fermentation medium: sucrose 45 g/L, peptone 40 g/L, MgSO₄·7H₂O 3.5 g/L, K₂HPO₄·3H₂O 3 g/L, KH₂PO₄ 1 g/L. The culture was carried out in 50 mL of medium in a flask at 220 rpm, with an initial pH of 6.0, an inoculum size of 4% (v/v), a temperature of 30 °C, and a fermentation time of 60 h.

All the media were autoclaved at 115 °C for 30 min prior to incubation.

### 2.3 Taxonomic identification of microorganisms

The taxonomic identification of the strain was mainly carried out through phenotype and 16S rRNA gene sequence detection, as well as scanning electron microscopy (SEM).

The detected nucleotide sequences were subjected to homology search using BLASTn against the GenBank database. By comparing the obtained gene sequence with homologous DNA sequences stored in the NCBI database. The strain identification was completed by Shanghai Paisenuo Biotechnology Co., Ltd.

SEM images of the microorganisms were captured using a high-resolution field-emission scanning electron microscope (FESEM; SU8020, Hitachi, Japan) operated at 10 kV. The samples were prepared according to previous reports (Cao et al., 2020).

### 2.4 Measurement of anticoagulant activity

The anticoagulant activity assay was based on previous reports with some modifications (Guangyu, 2003; Wei et al., 2011). Thrombin (10 NIH/mL) and fibrinogen (0.2%, w/v) were each dissolved in Tris–HCl buffer (50 mM, pH 7.4) containing 0.154 mM NaCl. In a microplate well, 40 μL of sample or control (Tris–HCl buffer) was mixed with 20 μL of thrombin, followed by the addition of 140 μL of fibrinogen. The resulting mixture was vigorously mixed for 10 s and incubated at 37 °C for 5 min. The optical density (OD) was then measured at 405 nm. Each experiment was performed in quadruplicate. In this assay, one unit (U/mL) of anticoagulant activity (defined as the inhibition of fibrinogen-induced coagulation) was defined as a change of 0.01/min in OD at 405 nm. The anticoagulant activity was calculated based on Equation (1).

$$A=(C-S)/(5^{*}\;0.01)$$ (1)

where A, C, S represent the anticoagulant activity, the OD of the control (Tris–HCl buffer), the sample, respectively.

### 2.5 Purification of anticoagulant protein derived from marine microorganism

The separation and purification of anticoagulant proteins were carried out following the sequential procedures:

(1) Ammonium sulfate fractionation: To determine the optimal saturation for fractionation, solid ammonium sulfate was added to the fermentation supernatant to achieve final saturations of 20, 30, 40, 50, 60, 70, and 80%, respectively. After ammonium sulfate addition, the mixtures were allowed to stand for 12 h at 4 °C. Each mixture was then centrifuged at 13,523 × g for 15 min at 4 °C. The anticoagulant activity of each resulting supernatant was measured.
(2) Filtration and concentration: The aforementioned solution was centrifuged at 4 °C and 13,523 × g for 15 min. After discarding the precipitate, the supernatant solution was processed using a tangential flow membrane filtration system (12 k ~ 14 kDa, GE, Amersham, Westborough, MA, United States) with Solution A for 12 h of filtration and concentration.
(3) Anion exchange chromatography: The dialysate was loaded onto an anion exchange column (DEAE Sepharose Fast Flow, Cytiva, United States) pre-balanced with Solution A. After complete ion exchange with the column, continuous gradient elution (0–100%) was performed using Solution B. The eluate was collected in a secondary collection tube.
(4) Gel filtration chromatography: The eluate with anticoagulant activity was collected, dialyzed using buffer Solution A1, and then loaded onto the Sepharose CL-6B (Cytiva, United States) column. Subsequently, it was eluted with buffer Solution B1, and the eluate was collected.
(5) Sodium dodecyl sulfate-polyacrylamide gel electrophoresis (SDS-PAGE) (Laemmli, 1970): Finally, the collected solution exhibiting anticoagulant activity was analyzed by SDS–PAGE using a 5% stacking gel and a 14% separating gel for identification.

### 2.6 Analysis of anticoagulant protein molecular weight

The molecular weight of the anticoagulant protein was estimated by high-performance liquid chromatography (HPLC) using a gel filtration column under native conditions (Irvine, 1994).

Chromatographic separation was carried out on a COSMOSIL 5Diol-120-II column (7.5 mm internal diameter × 300 mm, Nacalai Tesque, Japan) using an HPLC system. The column was equilibrated and eluted with 0.05 M sodium phosphate buffer (pH 6.8) at 30 °C with a flow rate of 1.0 mL/min.

To generate a calibration curve, the column was calibrated with a set of standard proteins of known molecular weights: phosphorylase b (97.4 kDa), ovalbumin (44.3 kDa), α-Chymotrypsinogen A (25.6 kDa), cytochrome C (12.4 kDa) and BSA (67 kDa). Each standard protein was individually dissolved in the mobile phase and injected under the same chromatographic conditions. The elution time for each standard was recorded, and a calibration curve was constructed by plotting the logarithm (base 10) of the molecular weight (log_10_ MW) against the corresponding elution time.

The anticoagulant protein was subjected to gel filtration (COSMOSIL 5Diol-120-II column) under identical conditions. Based on its elution time, the molecular weight was calculated by interpolation from the calibration curve.

### 2.7 Detection of protein concentration

The protein concentration was determined using the Bradford method with BSA as the standard protein (Bradford, 1976).

0.5 mL of the sample was mixed with 2.5 mL of Coomassie Brilliant Blue G-250 solution, followed by incubation at 25 °C for 5 min. The absorbance values were measured using a microplate reader at a wavelength of 595 nm.

### 2.8 Physicochemical characterization of the anticoagulant protein

The optimal temperature of the anticoagulant protein was determined by measuring the anticoagulant activity at 25 °C, 30 °C, 37 °C, 45 °C, 50 °C, and 55 °C. To evaluate the thermal stability of the anticoagulant proteins, the proteins were incubated at the same temperature conditions (25 °C, 30 °C, 37 °C, 45 °C, 50 °C, and 55 °C) for 2 h, followed by the detection of the activity. The anticoagulant activity was expressed as 100% of the highest anticoagulant activity, and the activity of the anticoagulant proteins at various temperatures was represented by relative anticoagulant activity.

The optimal reaction pH was determined by measuring the anticoagulant activity under different pH conditions (pH 4.0–11.0). To evaluate the pH stability, the anticoagulant proteins were incubated under different pH conditions (pH 4.0–11.0) for 2 h, followed by determination of the activity. The anticoagulant protein activity at each pH level was expressed as relative anticoagulant activity, with the highest anticoagulant activity set as 100%.

Different metal ions (Zn^2+^, Cu^2+^, Fe^3+^, Mn^2+^, Ca^2+^) were added to the sample at concentrations of 5 mM and 10 mM, respectively, and the mixture was incubated in 0.02 M PBS buffer (pH 7.0) at 4 °C for 2 h to evaluate their effects on anticoagulant activity.

### 2.9 Hemolytic safety evaluation of the anticoagulant protein

#### 2.9.1 Hemolysis test

Based on previously reported methods with minor modifications (Zhao et al., 2013), Columbia blood agar plates supplemented with 7% (v/v) defibrinated goat blood were used. Holes 3 mm in diameter were punched into the agar, and aliquots of anticoagulant proteins obtained during purification were added to each well. Physiological saline was added as a negative control. The plates were incubated at 37 °C for 12 h, after which the formation of a clear zone around each well was evaluated.

#### 2.9.2 Assessment of hemolysis rate

Based on previously reported methods with minor modifications (Rastogi et al., 2012), a 3.8% sodium citrate solution was added to bovine blood at a ratio of 1:9 (v/v). The mixture was centrifuged at 845 × g for 5 min, after which the supernatant was removed, and the remaining red blood cells (RBCs) were washed three times with normal saline.

The washed RBCs were mixed with electrophoretically purified anticoagulant protein at various concentrations (50, 5, and 2.5 μg/mL) at a ratio of 3:2 (v/v) and incubated at 37 °C for 1 h. Double-distilled water was used as the positive control, and normal saline (0.9% NaCl) served as the negative control. Following incubation, the samples were centrifuged at 845 × g for 5 min. The supernatant was collected, and its absorbance was measured at 540 nm to calculate the hemolysis rate.

The hemolysis rate (%) was calculated as Equation (2) (Administration, 2006):

(ODsample−ODnegative)/(ODpositive−ODnegative)×100% (2)

### 2.10 Effect of anticoagulant on erythrocyte aggregation

Following previously reported methods with minor modifications (Jiejing, 2008), an anticoagulant protein purified to electrophoretic homogeneity was used to assess its effect on erythrocyte aggregation.

Erythrocyte suspensions were prepared from bovine anticoagulated blood by centrifugation at 376 × g for 5 min, followed by removal of the supernatant. The pelleted cells were washed three times with physiological saline until the supernatant became clear and colorless, and then resuspended in saline to a final concentration of 2% (v/v). Then, a 2% RBCs suspension was gently mixed with the electrophoretically purified anticoagulant protein solution at concentrations of 50, 25, and 12.5 μg/mL at a volume ratio of 1:4 (RBCs to protein solution). For each concentration, separate samples were then incubated at 37 °C for 30 min and 120 min, respectively. After incubation, RBCs morphology was immediately observed under an optical microscope at 40 × magnification. Physiological saline was used as a negative control.

### 2.11 Anticoagulant effect testing

The assay was established based on the principle that thrombin converts fibrinogen into an insoluble fibrin clot (Kita et al., 2002). Four microcentrifuge tubes were prepared for the assay as follows: each tube received 120 μL of thrombin solution (10 NIH/mL). Subsequently, 240 μL of anticoagulant protease solution at concentrations of 5 μg/mL and 2.5 μg/mL were added to two separate tubes. The third tube, serving as a negative control, received 240 μL of physiological saline, while the fourth tube, as a positive control, received 240 μL of heparin sodium solution (2,000 μg/mL). All tubes were mixed well. After preheating in a 37 °C water bath for 5 min, 840 μL of pre-warmed fibrinogen solution (0.2%) was added to each tube. The mixtures were thoroughly mixed and incubated further in the 37 °C water bath for 15 min. Following incubation, fibrin formation was observed.

### 2.12 Fibrinogen degradation assay

The fibrinogen degradation assay was conducted based on the method described by Rastogi et al. with minor modifications (Rastogi et al., 2012), as outlined below. Fibrinogen (0.2%, w/v) was uniformly mixed with 5 μg/mL of the electrophoretically purified anticoagulant protein and incubated at 37 °C for 3 h. Samples were collected every 30 min during the incubation period. Following collection, each sample was mixed with loading buffer at a ratio of 1:3 (v/v) and heated in boiling water for 10 min to terminate the reaction. Subsequently, the degradation of fibrinogen was assessed using 14% SDS-PAGE. A system consisting of fibrinogen mixed with 0.9% sodium chloride (NaCl) saline served as the control.

### 2.13 Statistical analysis

All experiments were performed at least three times. The major data were analyzed using Origin 9.0. The size of RBCs was measured from microscopic images with ImageJ 1.52a software. Statistical analyses were performed using GraphPad Prism software (version 8.4.2; GraphPad Software, United States). The groups were compared using oneway analysis of variance (ANOVA). *p* < 0.05 was considered significant. All data are presented as the mean ± standard deviation (SDs).

## 3 Results

### 3.1 Taxonomic identification of microorganisms

**Figure 1:**
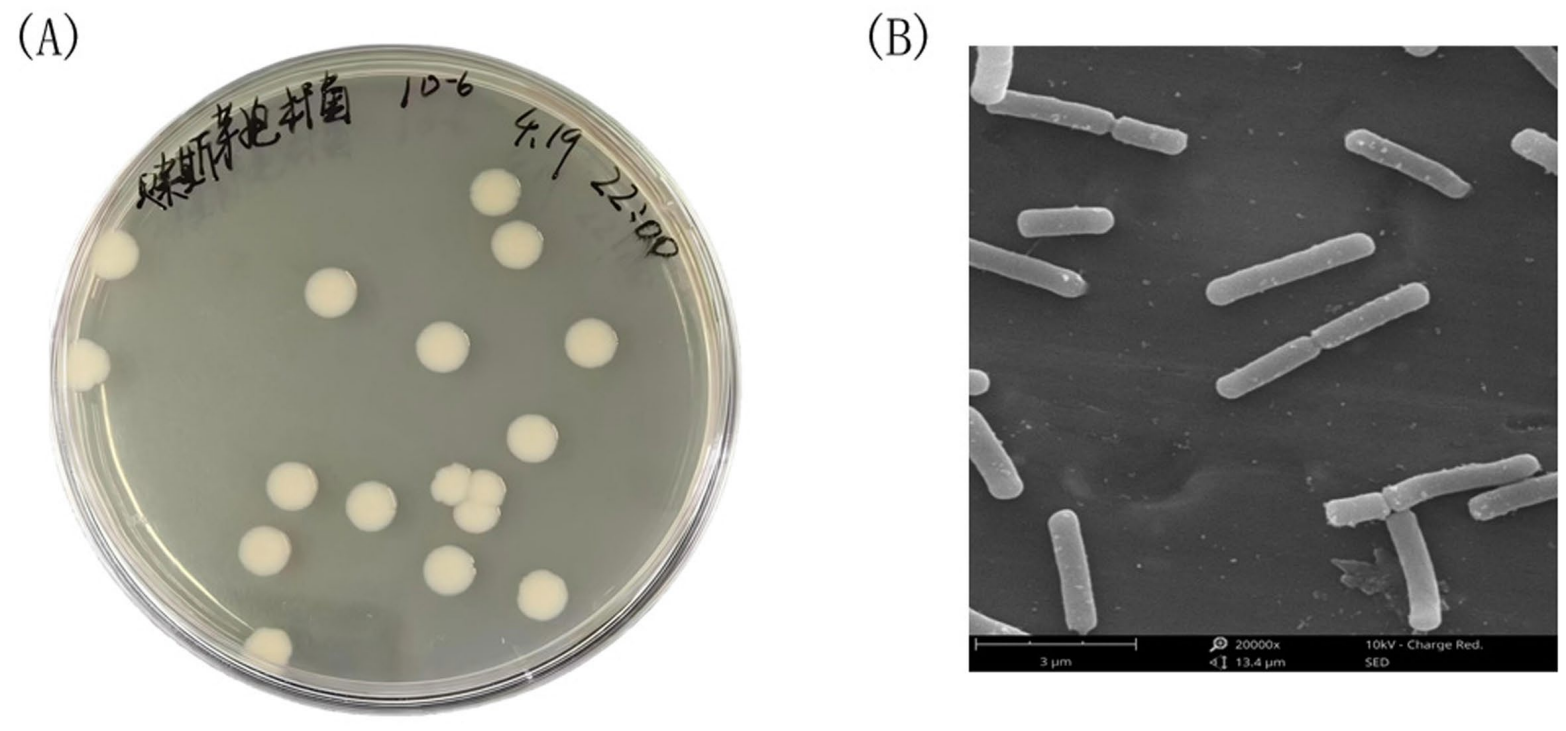
Taxonomic classification and identification of the marine microorganisms. **(A)** Colony morphology of the strain on LB agar plates; **(B)** The morphology of marine microorganisms was observed under a scanning electron microscope (SEM) at 20,000× magnification.

Colonies of marine microorganisms cultured on the Luria-Bertani (LB) plates were round, white, moist and smooth-edged (Figure 1A). The strain was observed to be rod-shaped by scanning electron microscopy (SEM) (Figure 1B).

The 16S rRNA gene sequence (No. PZ293158) obtained from the strain B01 was subjected to BLAST analysis against the NCBI database. The results revealed that the highest homology was observed between the strain and the sequence of *Bacillus velezensis*, which was consequently selected for species identification.

### 3.2 Purification and identification of the anticoagulant protein

**Figure 2:**
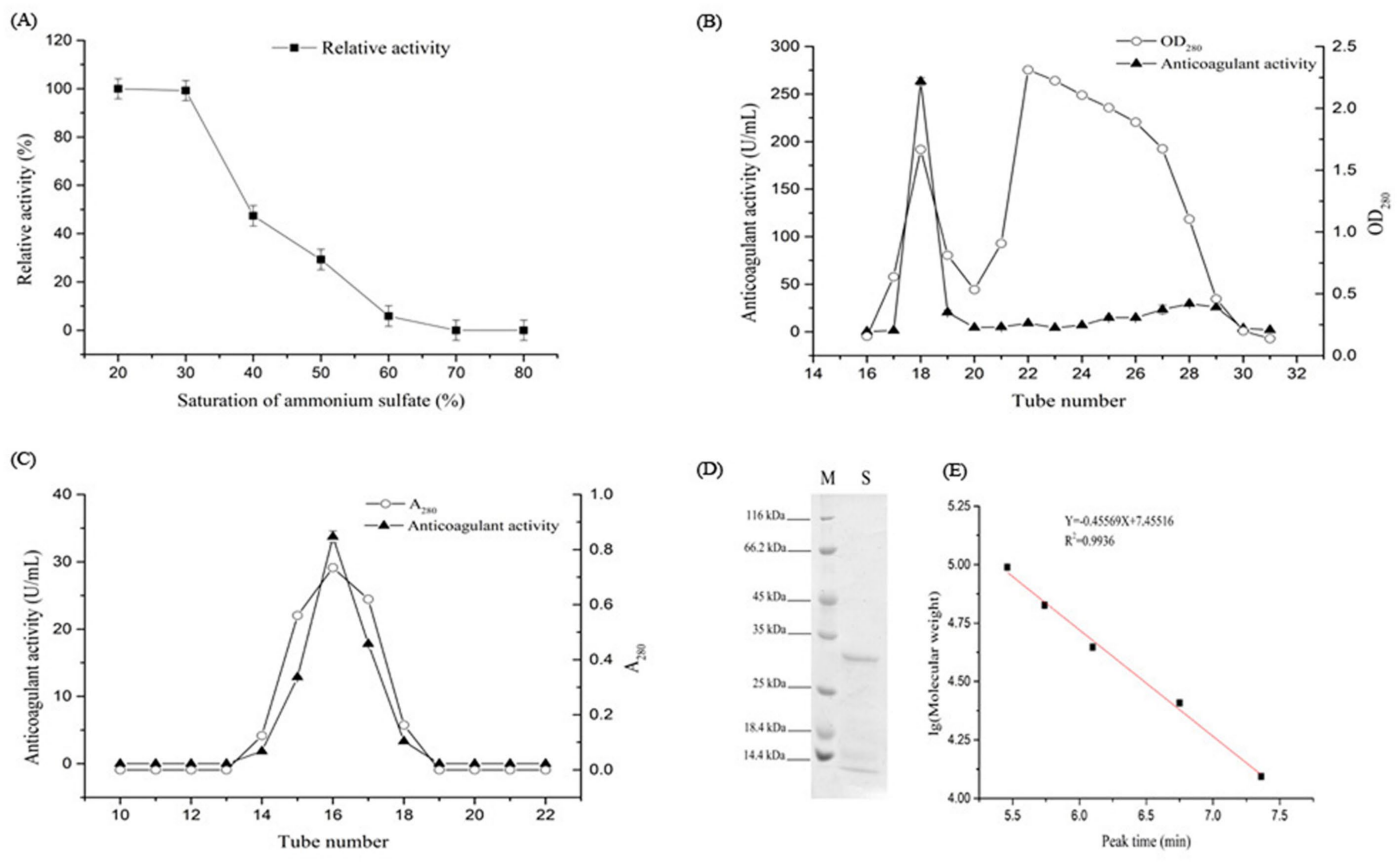
Purification and identification of anticoagulant protein. **(A)** Settlement curve of ammonium sulfate saturation; **(B)** Anion exchange chromatography; **(C)** Gel filtration chromatography; **(D)** SDS-PAGE electrophoresis of the anticoagulant protein (M represents protein molecular weight marker; S represents the sample of anticoagulant protein); **(E)** Peak time curve of protein standard.

Ammonium sulfate fractionation was first performed to enrich the anticoagulant protein from the fermentation supernatant. As shown in Figure 2A, the anticoagulant activity in the supernatant remained relatively stable at ammonium sulfate saturations below 30%, indicating that most contaminating proteins precipitated within this range. When the saturation was increased to 70%, the activity in the supernatant decreased to nearly undetectable levels, suggesting that the target protein was completely precipitated by 70% saturation. Based on these results, a two-step fractionation protocol was established: the 30% saturation precipitate was discarded to remove impurities, and the 30–70% saturation precipitate was collected for further purification. Then, The crude protein obtained from ammonium sulfate fractionation was subsequently loaded onto a DEAE-Sepharose Fast Flow anion exchange column. As shown in Figure 2B, two major protein peaks were eluted from the column (fractions 18 and 22). Fractions were collected based on absorbance at 280 nm and assayed for anticoagulant activity. High activity was detected in fraction 18, whereas no activity was detected in other fractions. The collected solution (containing 10.64 mg of anticoagulant protein) will be used for subsequent experiments. As presented in Figure 2C, a single symmetrical protein peak was eluted, and anticoagulant activity was detected exclusively in fraction 16, which coincided precisely with the absorbance peak. This result indicated that the target protein had been successfully purified to homogeneity.

The anticoagulant protein was purified using ammonium sulfate precipitation, DEAE-Sepharose anion exchange chromatography, and Sepharose CL-6B gel filtration chromatography, resulting in substantial improvements in specific activity and purity. Following ammonium sulfate precipitation of fermentation broth, the specific activity increased by 1.59-fold, with a protein recovery rate of 81.76%. After anion exchange and Sepharose CL-6B gel filtration chromatography, the final specific activity reached 2792.27 U/mg, representing a 48.85-fold increase compared to the fermentation broth (Table 1).

**Table 1:** Steps of the purification process of anticoagulant protein from *Bacillus velezensis.*

Purification step	Total protein (mg)	Total activity (U)	Specific activity (U/mg)	Purification fold	Recovery (%)
Crude extract	603.57	34,500	57.16	1	100.00
Ammonium sulfate	310.38	28207.2	90.88	1.59	81.76
DEAE-Sepharose	10.64	17236.2	1618.77	28.32	49.96
Sepharose CL-6B	0.89	2487.45	2792.27	48.85	7.21

The anticoagulant protein collected from Sepharose CL-6B gel filtration chromatography was analyzed by SDS-PAGE, and the results are shown in (Figure 2D). A single protein band was observed, corresponding to a molecular weight ranging from 25 kDa to 35 kDa. Using standard protein samples with known molecular weights, their elution times on a COSMOSIL 5Diol-120-II gel column were determined. The molecular weights and corresponding elution times of each standard protein are summarized in Table 2. A standard curve was constructed by plotting elution time on the x-axis against the logarithm of molecular weight (log value) on the y-axis, as shown in Figure 2E. The curve was fitted to Equation (3):

$$Y=-0.4556\mathrm{9X}+7.45516\;(R^{2}=0.9936)$$ (3)

**Table 2:** Peak time of protein standard.

Protein standard	Molecular weight (kDa)	lg (molecular weight)	Peak time (min)
Phosphorylase b	97.4	4.99	5.46
BSA	67	4.83	5.74
Ovalbumin	44.3	4.65	6.10
α-Chymotrypsinogen A	25.6	4.41	6.75
Cytochrome C	12.4	4.09	7.36

Based on the elution time of 6.63 min for the anticoagulant protein, its molecular weight was calculated to be 27.2 kDa using the above equation.

### 3.3 Physicochemical properties of the anticoagulant protein

**Figure 3:**
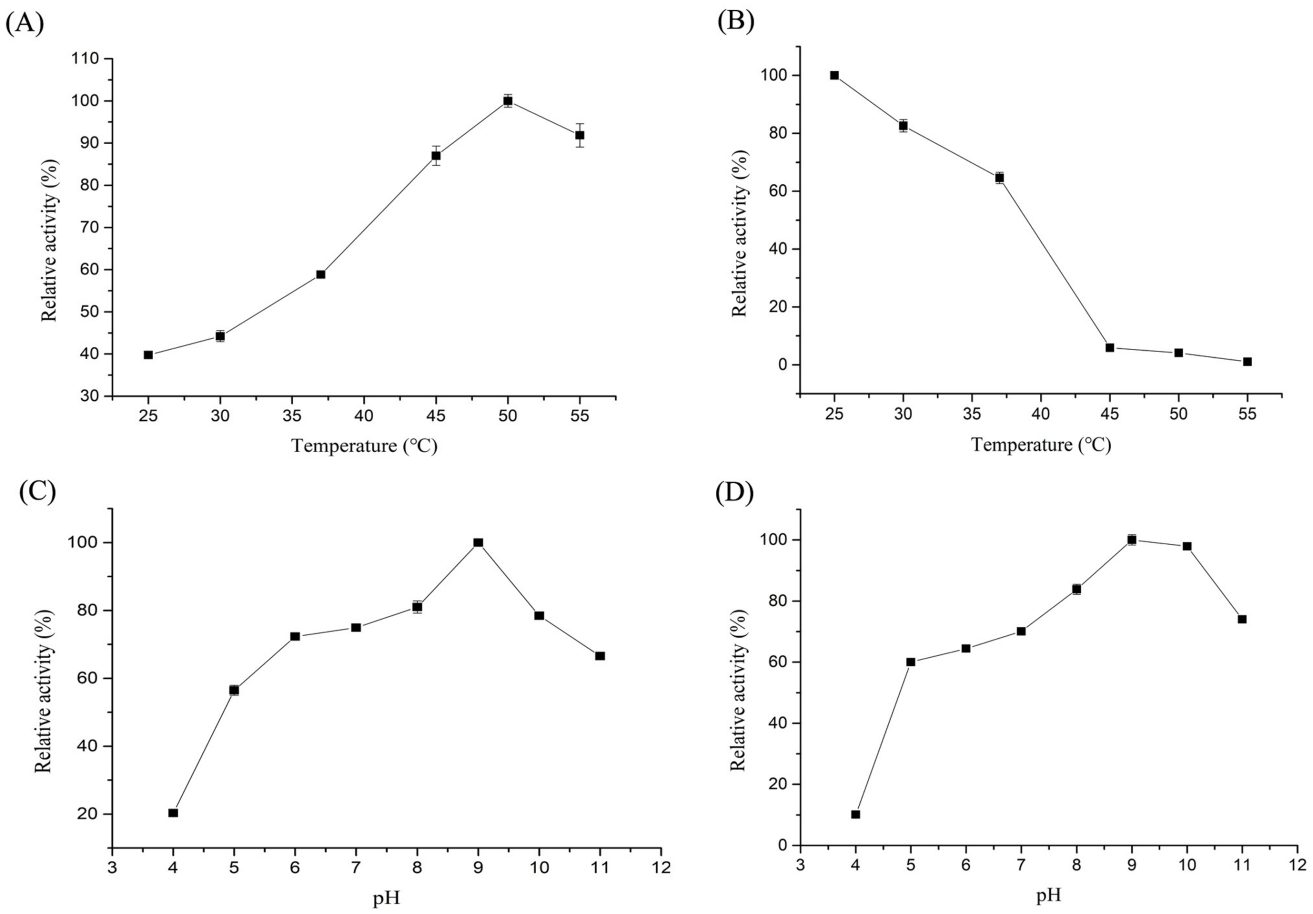
Effect of temperature and pH on anticoagulant protein activity and stability: **(A)** Optimal temperature; **(B)** temperature stability; **(C)** optimal pH; **(D)** pH stability.

The anticoagulant activity of the proteins was assessed following incubation at varying temperatures. The optimal reaction temperature was determined to be 50 °C. Relative anticoagulant activity decreased when the temperature exceeded 50 °C, although it remained high at 55 °C (91.85%) (Figure 3A). Following a 2-h incubation at different temperatures, the anticoagulant proteins exhibited favorable stability within the range of 25 °C to 37 °C (Figure 3B), with the highest activity recorded at 25 °C. Relative activity progressively declined as the temperature increased, reaching 64.59% at 37 °C and dropping sharply to 5.86% at 45 °C. These results suggest that elevated temperatures induce protein denaturation and inactivation, thereby reducing stability (Wang et al., 2007). Accordingly, higher temperatures are unfavorable for the preservation of anticoagulant proteins.

The relative anticoagulant activity of the anticoagulant protein in different pH buffer solutions is presented in Figure 3C, with an optimal pH 9. When the pH ranged from 4 to 7, the relative anticoagulant activity remained below 80%, with the lowest activity observed at pH 4 (20.31%). As the pH increased from above 7 to 9, the relative activity correspondingly increased. These results indicate that the anticoagulant protein exhibits favorable activity under alkaline conditions. As shown in Figure 3D, the anticoagulant protein maintained high activity levels in alkaline environments (pH 8–11) after 2 h at 25 °C, with particularly good stability observed in the pH 9–10 range. In contrast, its stability under acidic conditions was markedly reduced, with relative activity falling below 10.15% at pH values lower than 4.

**Figure 4:**
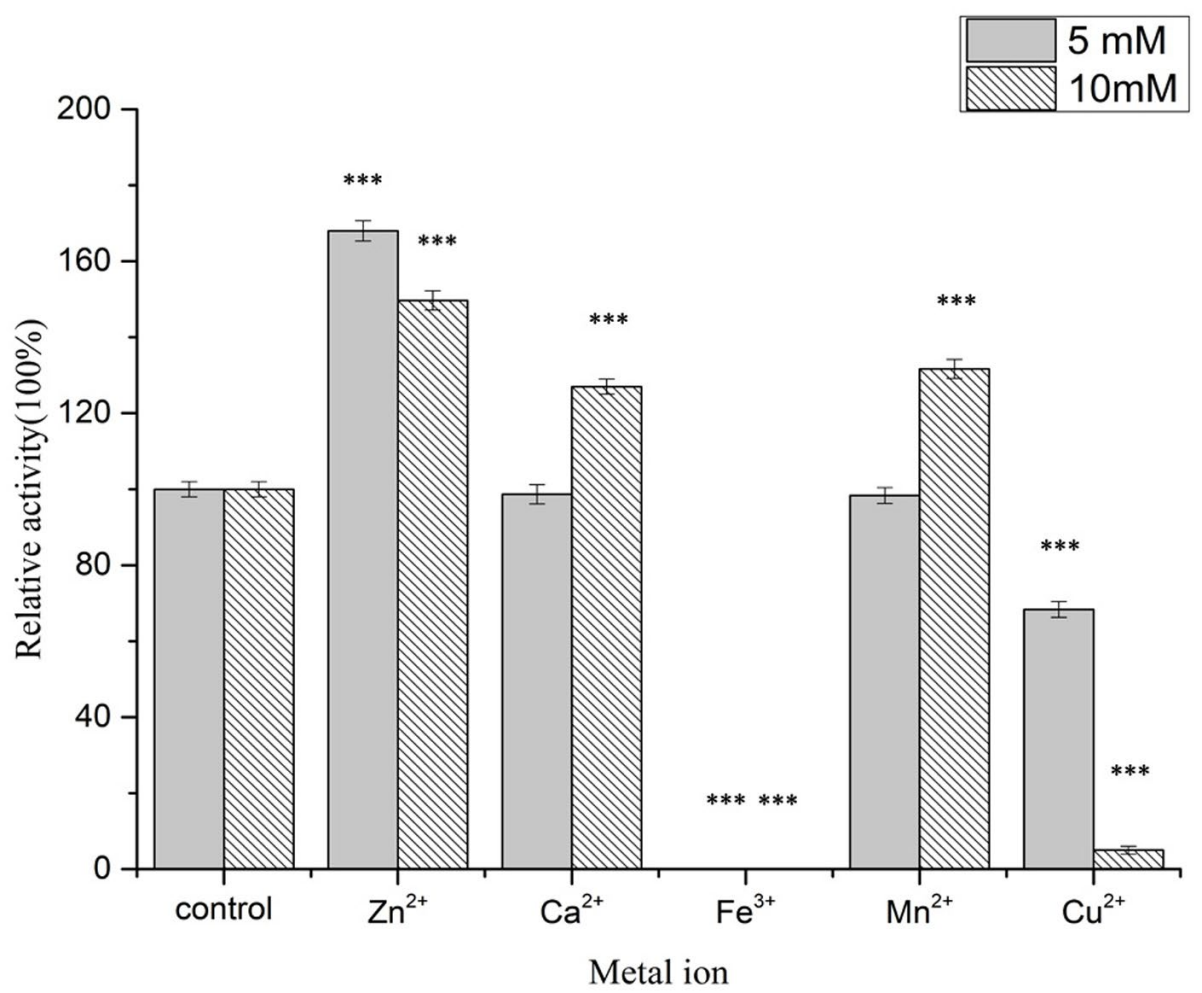
Effect of metal ions on the activity of anticoagulant protein. Data are presented as mean ± SD (*n* = 3). Asterisks indicate significant differences compared with the control group at the same concentration (***p < 0.001).

Relative anticoagulant activity was assessed at metal ion concentrations of 5 mM and 10 mM, with the activity of the control group set as 100% (Figure 4). At a concentration of 5 mM, Zn^2+^ significantly enhanced anticoagulant activity by 40.49% compared with the control group (*p* < 0.001), whereas Cu^2+^ markedly inhibited it (*p* < 0.001). Fe^3+^ completely suppressed the activity of the anticoagulant protein (*p* < 0.001), while Ca^2+^ and Mn^2+^ exhibited no significant effect. These results indicate that the anticoagulant protein is highly sensitive to Zn^2+^ and Fe^3+^, with even low concentrations capable of significantly affecting its activity.

At a metal ion concentration of 10 mM, the Zn^2+^ treatment group exhibited the highest relative anticoagulant activity, with an increase of 33.17% compared with the control group (*p* < 0.001). Mn^2+^ and Ca^2+^ also enhanced anticoagulant activity, showing increases of 21.18 and 18.09%, respectively (*p* < 0.001). In contrast, Cu^2+^ significantly inhibited the activity of the anticoagulant protein, resulting in a 63.43% decrease relative to the control group (*p* < 0.001). These results indicate that Cu^2+^ and Fe^3+^ exert strong inhibitory effects on the anticoagulant protein, whereas Zn^2+^, Mn^2+^, and Ca^2+^ demonstrate positive promoting effects. Notably, Zn^2+^ increased anticoagulant activity by 40.49% at 5 mM and by 33.17% at 10 mM.

### 3.4 Hemolytic safety assessment

**Figure 5:**
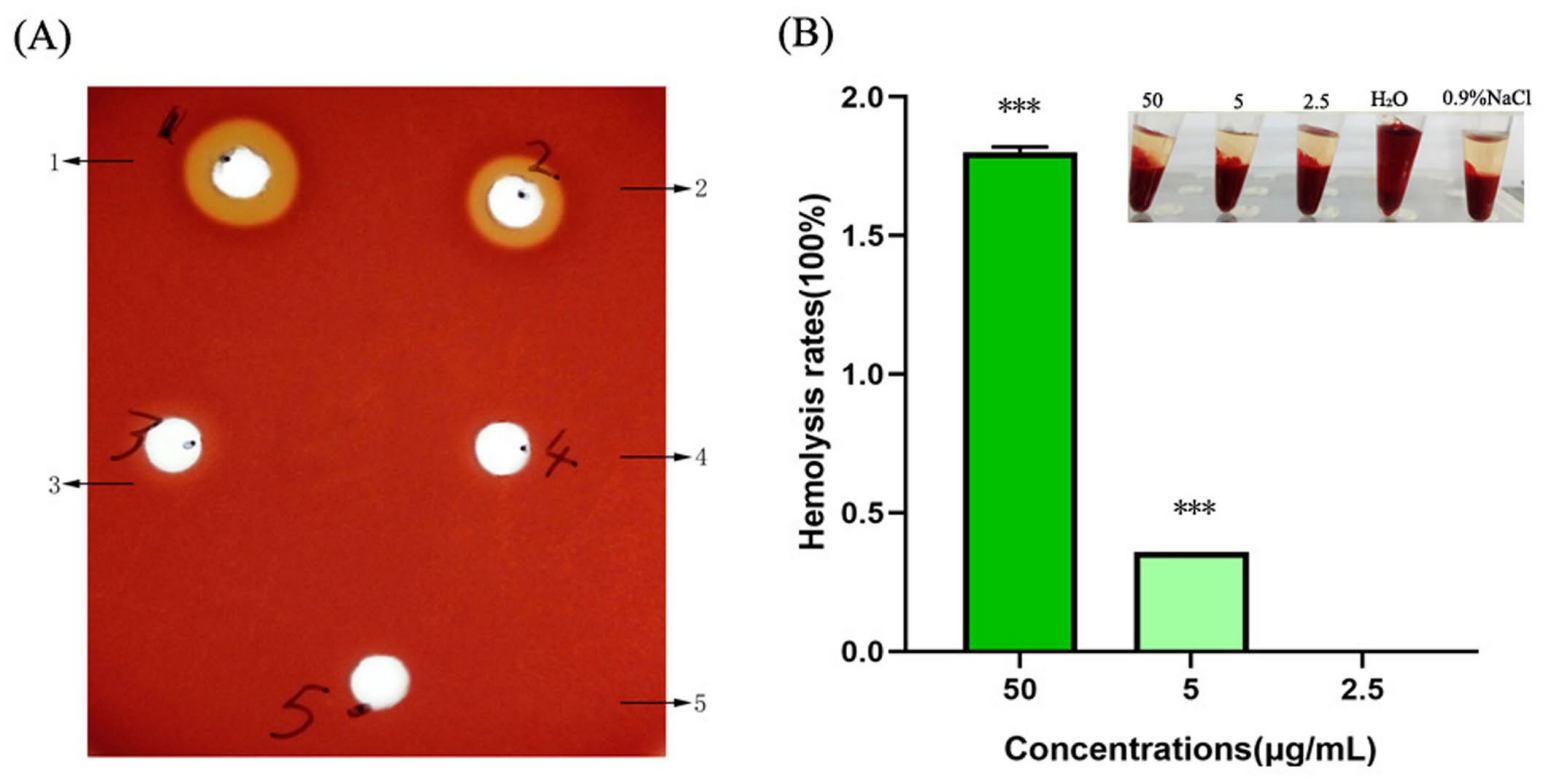
Hemolytic safety assessment. **(A)** Hemolysis effect of anticoagulant protein on blood agar plate. Numbers 1–5 correspond to: (1) fermentation broth; (2) ammonium sulfate precipitate; (3) anticoagulant protein purified by DEAE Sepharose Fast Flow; (4) anticoagulant protein purified by Sepharose CL-6B; and (5) physiological saline (negative control). **(B)** Hemolysis rate of RBCs after incubation with different samples. Samples 1–3: electrophoretically pure anticoagulant protein at concentrations of 50, 5, and 2.5 μg/mL; sample 4: double-distilled water; sample 5: normal saline. Data are presented as mean ± SD (*n* = 3). Asterisks indicate significant differences compared with the negative control (normal saline, 0%) (***p < 0.001).

Blood agar plate assay showed that both the fermentation broth and the ammonium sulfate-precipitated protein formed clear zones, indicating RBCs degradation and a potential hemolytic risk. The DEAE-purified anticoagulant protein displayed weak hemolytic activity, whereas the electrophoretically pure protein, like the saline control, produced no clear zones (Figure 5A).

Electrophoretically purified anticoagulant protein, double-distilled water, and physiological saline were each mixed with RBCs, incubated at 37 °C for 1 h, and then centrifuged at 845 × g for 5 min. The supernatant was collected, and its absorbance was measured by the turbidimetric method at 540 nm (OD₅₄₀). The results showed that at anticoagulant protein concentrations of 50 μg/mL, 5 μg/mL, and 2.5 μg/mL, the hemolysis rates were (1.80 ± 0.02)%, (0.36 ± 0.00)%, and 0, respectively. The hemolysis rate in the double-distilled water group was 100% (positive control), while no hemolysis was observed in the physiological saline group (negative control) (Figure 5B). Compared with the negative control, the hemolysis rates in the 50 μg/mL group (1.80% ± 0.02%) and the 5 μg/mL group (0.36% ± 0.00%) were significantly increased (*p* < 0.001), whereas the 2.5 μg/mL group (0%) showed no statistically significant difference. These findings indicate that the anticoagulant protein has an acceptable safety profile toward RBCs and holds potential as a therapeutic agent for thrombotic diseases.

### 3.5 Effect of anticoagulant on erythrocyte aggregation

**Figure 6:**
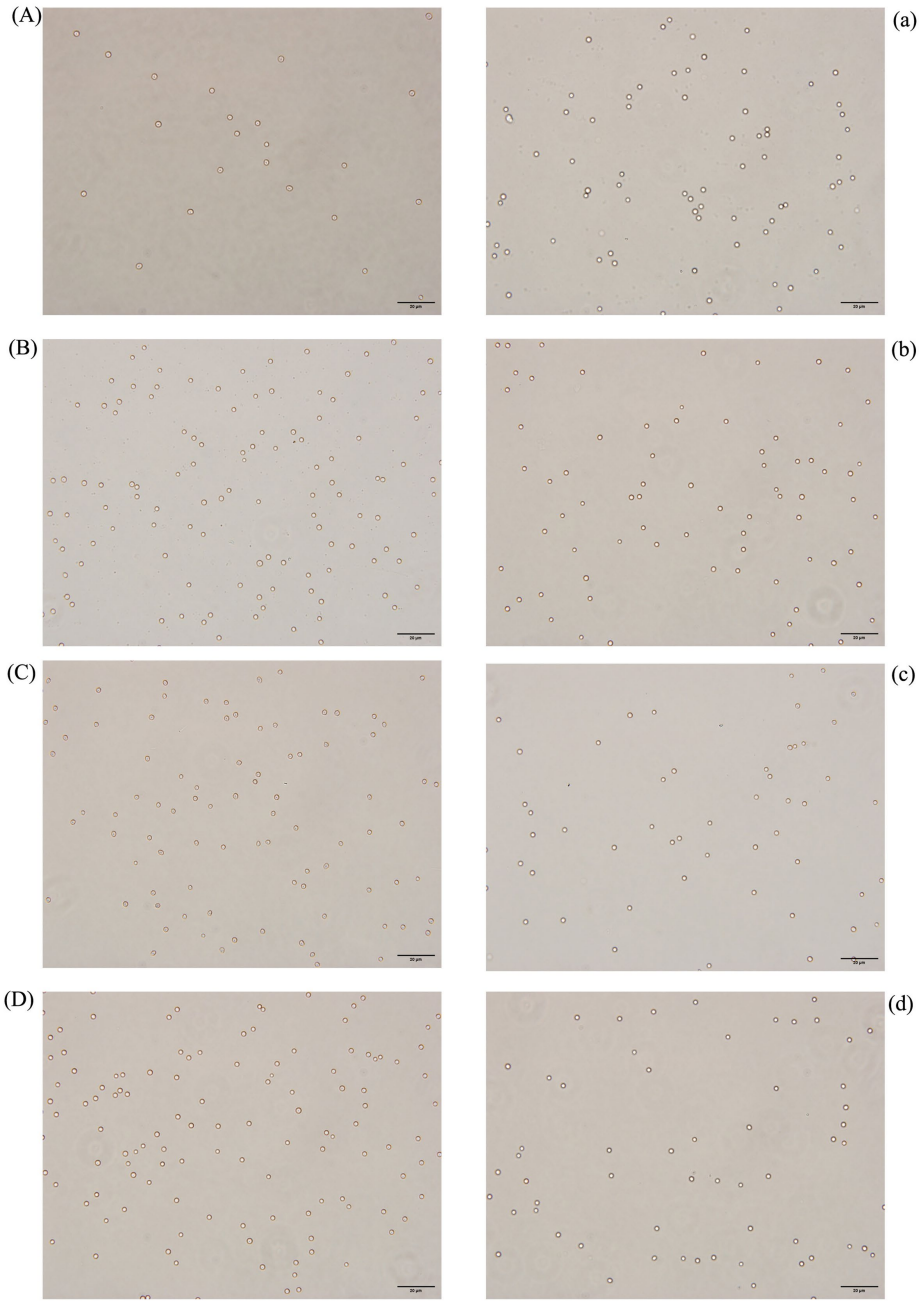
Effect of anticoagulant on erythrocyte aggregation. **(A–D)** and a–d indicate the aggregation status of samples and RBCs after incubation at 37 °C for 30 min and 120 min, respectively. **(A)** and **(a)** represent the saline control group; **(B)** and **(b)**, **(C)** and **(c)** and **(D)** and **(d)** correspond to anticoagulant proteins with anticoagulant activities of 50, 25, and 12.5 μg/mL, respectively.

The aggregation status of samples after 30 min of incubation with RBCs is denoted as A–D, and that after 120 min as a–d (Figure 6). When physiological saline was mixed with RBCs at a ratio of 1:4, the RBCs remained intact and well dispersed throughout the 120-min incubation period, with no evidence of aggregation. Similarly, treatment with anticoagulant proteins at concentrations of 50, 25, and 12.5 μg/mL did not induce RBC aggregation, irrespective of the incubation duration (30 or 120 min). In all cases, RBCs were well dispersed and morphologically intact, showing no significant difference from the physiological saline control group. These results indicate that the anticoagulant proteins do not trigger RBC aggregation or cause cellular damage.

### 3.6 Anticoagulant effects

**Figure 7:**
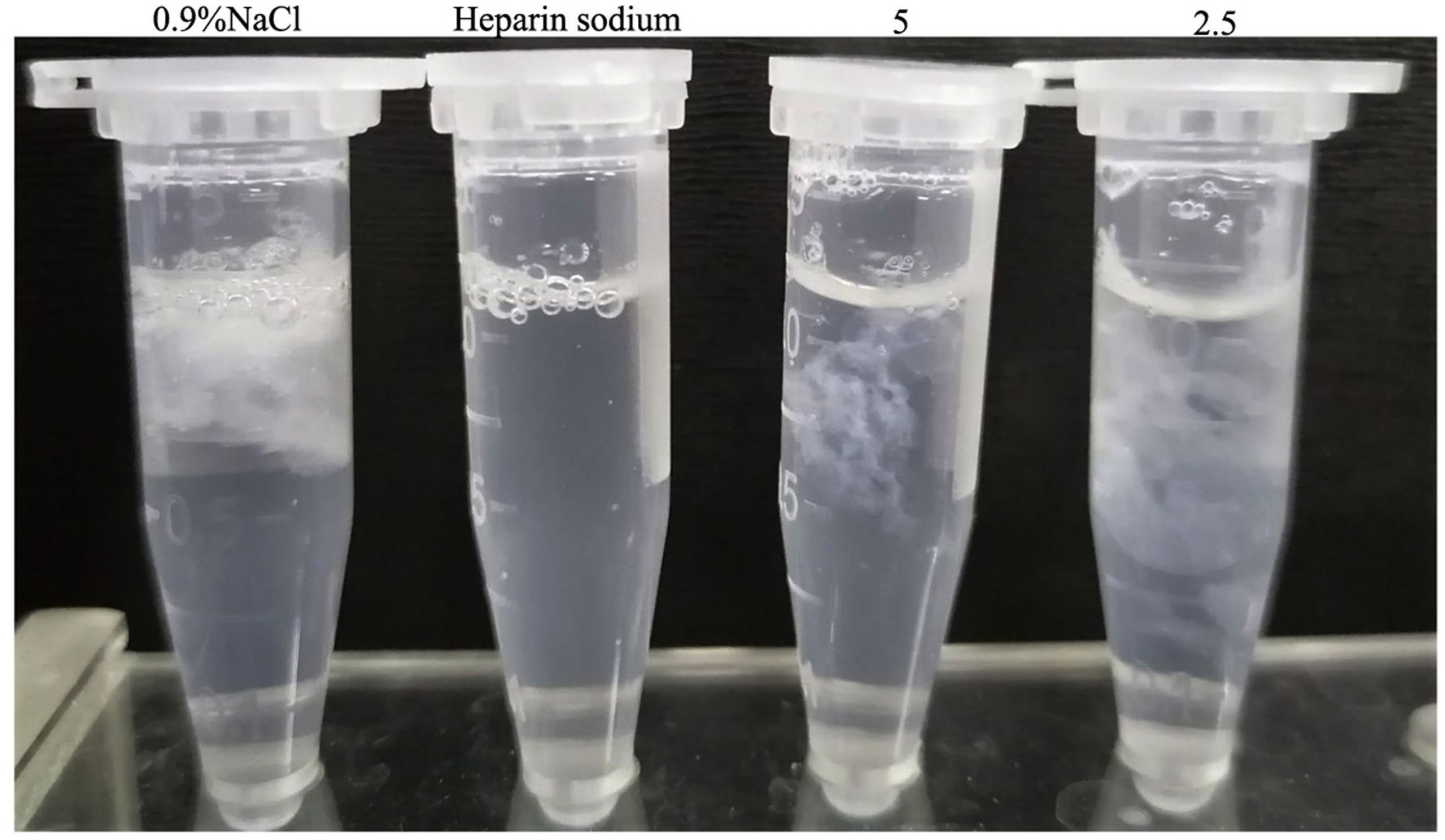
External anticoagulant effects (formation of fibrin). Lanes 1–4 correspond to physiological saline, heparin sodium (2,000 μg/mL), and anticoagulant protein at 5 μg/mL and 2.5 μg/mL, respectively.

As shown in Figure 7, the negative control group (physiological saline) produced the largest amount of fibrin, whereas no fibrin was formed in the positive control group (heparin sodium). In the experimental groups treated with the anticoagulant protein, fibrin was detected in all test tubes. However, fibrin production decreased as the concentration of the anticoagulant protein increased. Thus, anticoagulant proteins suppress fibrin generation and exert anticoagulant effects.

### 3.7 Degradation of fibrinogen

**Figure 8:**
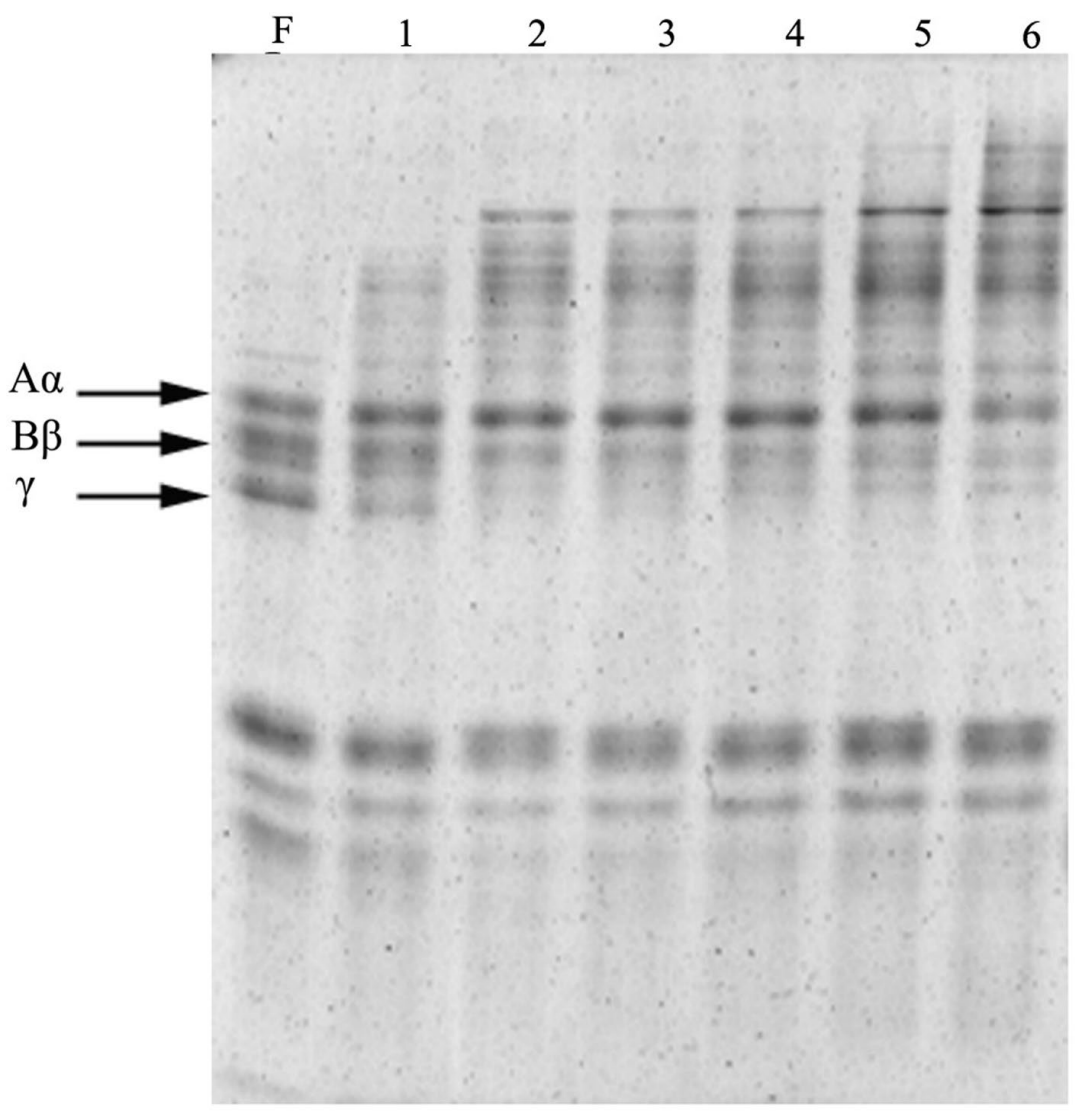
SDS-PAGE analysis of time-dependent fibrinogen degradation. Lane F, control group (0.9% NaCl, 37 °C, 0 min); lanes 1–6, samples co-incubated with the anticoagulant protein for 30, 60, 90, 120, 150, and 180 min, respectively.

After degradation treatment, fibrinogen was analyzed by SDS-PAGE, which revealed three major bands corresponding to the Aα, Bβ, and γ chains (Figure 8, lane F). Following 30 min of incubation, the γ chain began to degrade (lane 1); by 60 min, it was almost completely degraded, exhibiting the fastest degradation rate among the three chains. Concurrently, the Bβ chain also showed substantial degradation at 60 min (lane 2). In contrast, the Aα chain was the most resistant to degradation, with noticeable degradation observed only after 180 min (lane 6). These results indicated that with prolonged incubation, the γ chain was degraded first, followed by the Bβ chain, and the Aα chain last. The γ and Bβ chains were more susceptible to degradation by anticoagulant proteins than the Aα chain.

## 4 Discussion

In this study, a strain capable of producing an anticoagulant protein was isolated from mud sediment collected from the South China Sea. Based on sequencing and sequence alignment of the 16S rRNA gene, combined with physiological, biochemical, and phenotypic analyses, the strain was identified as *Bacillus velezensis* and designated *Bacillus velezensis* B01. *Bacillus velezensis* has been widely applied in biological control and agriculture (Wang J. et al., 2023), including the management of plant root rot (Wang C. et al., 2023) and bacterial infections (Zhao et al., 2024). Moreover, it has demonstrated potential as a probiotic in animal studies, particularly in antibacterial and immune-enhancing applications (Yi et al., 2018). However, reports on the production of anticoagulant substances by *Bacillus velezensis* remain limited.

After purification, a homogeneous anticoagulant protein was obtained with a recovery rate of 7.21% and a 48.85-fold increase in specific activity. Notably, significant loss of anticoagulant activity occurred during the gel filtration (Sepharose CL-6B) step, which was speculated to result from nonspecific adsorption of the protein to the Sephacryl gel. Nevertheless, this step still facilitated impurity removal and enhanced the specific activity of the protein. SDS-PAGE and gel filtration analyses revealed that the molecular weight of the anticoagulant protein was approximately 27.2 kDa. To date, few reports have described the molecular weight of anticoagulant substances derived from *Bacillus* species; however, existing studies have shown that a fibrinolytic enzyme derived from *Bacillus cereus* exhibits anticoagulant effects with a molecular weight of 39.5 kDa (Majumdar et al., 2015), and nattokinase derived from *Bacillus subtilis* exhibits antithrombotic effects with a molecular weight of 28 kDa (Kapoor et al., 2015), which is comparable to that of the anticoagulant protein reported herein.

The observed discrepancy between the optimal reaction temperature (50 °C) and the moderate thermal stability at physiological temperature (37 °C, with 65% residual activity after 2 h) warrants careful consideration regarding the potential clinical translation of this anticoagulant protein. In a physiological setting, the therapeutic efficacy of an anticoagulant is determined not only by its peak activity but also by its functional half-life in the circulation. The observation that the protein retains approximately two-thirds of its activity after a 2-h incubation at 37 °C indicates that it is not rapidly denatured under body temperature conditions, a stability profile that compares favorably with other fibrinolytic enzymes currently under investigation (Sharma et al., 2021). Furthermore, many protein-based therapeutics with similar thermal stability characteristics have been successfully developed for clinical use, often with the aid of well-established formulation strategies. These include lyophilization with stabilizing excipients (Arun Butreddy et al., 2021), the addition of protective agents such as sugars, polyols, or amino acids (Manning et al., 2024; Castañeda Ruiz et al., 2022), or protein engineering approaches to enhance thermal tolerance (Ravichandran et al., 2025). Notably, similar stability challenges have been recognized for other microbial fibrinolytic enzymes, including nattokinase, where formulation and immobilization strategies have been actively pursued to overcome these limitations (Ashokan and Mohanasrinivasan, 2025). Therefore, while the current thermal stability profile may pose a challenge for long-term storage or sustained *in vivo* activity, it does not preclude further development. Future studies will focus on protein engineering and formulation optimization to improve the thermal robustness of this anticoagulant protein without compromising its unique substrate specificity toward the fibrinogen γ chain.

This study evaluated the impact of varying pH conditions on the relative activity of an anticoagulant protein, revealing a marked pH dependence. Under acidic conditions (pH 4–7), the protein’s activity decreased significantly, whereas under alkaline conditions (pH 8–11), it remained relatively high. Further pH stability analysis indicated that the protein was most stable within the pH range of 9–10. When the pH fell below 4, the relative activity dropped to less than 10.15%, suggesting that acidic environments may induce conformational changes or inactivation of the active center. This pH-dependent behavior may be closely related to the protein’s origin and evolutionary adaptation. The anticoagulant protein used in this study is derived from a marine strain of *Bacillus velezensis*, which has long thrived in a high-salt, slightly alkaline marine environment. Consequently, the protein exhibits high catalytic activity and structural stability under alkaline conditions. Similar observations have been reported for other marine microbial enzymes; for instance, proteases (Le Gia et al., 2014) and haloalkaline xylanase (Menon et al., 2010) produced by certain marine bacteria also show a preference for an alkaline pH range. In summary, the pH characteristics of this anticoagulant protein are the result of natural selection and evolutionary adaptation. From a pharmacological perspective, 70% residual activity at physiological pH does not necessarily preclude therapeutic development. The observed partial activity at pH 7.4 indicates that the protein is not inactivated under physiological conditions, but rather operates at a reduced catalytic efficiency compared with its maximal capacity. This is a manageable limitation rather than a fundamental barrier to clinical translation. Moreover, the pH profile of a therapeutic protein can be modulated through established strategies. Protein engineering approaches, particularly site-directed mutagenesis of residues that influence the ionization state of the active site, have been successfully employed to shift the pH optimum of proteases toward more physiologically compatible ranges (Kusano et al., 2006; Makde et al., 2006). Alternatively, formulation strategies that employ stabilizing excipients can enhance protein stability and activity under physiological pH conditions (Manning et al., 2024).

The regulatory effects of various metal ions on anticoagulant proteins exhibit marked selectivity and concentration dependence. Among these, Zn^2+^, Mn^2+^, and Ca^2+^ function as positive regulators, with Zn^2+^ demonstrating the most potent activating effect. In contrast, Cu^2+^ and Fe^3+^ act as strong inhibitors. Existing research has demonstrated that Zn^2+^ can function as an enzyme cofactor. By binding to amino acid side chains outside the active center, it forms a metal active site that stabilizes protein conformation or facilitates substrate binding, thereby enhancing enzyme activity (Qian et al., 2019). In the present study, the activating effect of Zn^2+^ exhibited a slight negative correlation with its concentration (5 mM being more effective than 10 mM), suggesting that excessively high concentrations may induce nonspecific binding or local structural perturbations, leading to reduced activation efficiency. Anticoagulant proteins exhibit high sensitivity to Cu^2+^ and Fe^3+^. These ions can disrupt the structure of the protein’s active center via redox reactions or chelation with key amino acid residues (e.g., cysteine and histidine), leading to irreversible inactivation.

The hemolytic safety of the electrophoretically pure anticoagulant protease from *Bacillus velezensis* B01 was evaluated. The results of the blood agar plate experiment showed that the electrophoretically pure protein did not form a transparent zone, indicating that the hemolytic activity in the crude sample was due to impurities rather than the target protease. The hemolysis rate assay results indicated that the hemolysis rate of the anticoagulant protein toward RBCs was significantly lower than the prescribed safety threshold of 5% (AAMI, 2017), suggesting that it did not damage the integrity of RBCs membranes within the expected concentration range. This may be attributed to the selective action of the anticoagulant protein on coagulation-related substrates, rather than to its recognition of RBCs membrane components. In contrast, reported microbial proteases, such as elastases derived from certain *Aeromonas* species or *Pseudomonas aeruginosa*, exhibit significant hemolytic activity due to their ability to hydrolyze RBCs membrane skeleton proteins (Nord et al., 1975). In summary, the anticoagulant protease obtained in this study possesses potential advantages in terms of functional specificity and membrane safety.

The anticoagulant protein did not induce RBCs aggregation at either 30 min or 120 min. The RBCs remained well dispersed with intact morphology, showing no significant difference from the saline control group. These results indicate that, within the tested concentration range, the anticoagulant protein did not cause significant hemolysis, aggregation, or severe morphological abnormalities in RBCs. This finding corroborates the conclusion of the hemolytic safety assessment, suggesting that the anticoagulant protein does not recognize or hydrolyze RBCs membrane proteins and that the intercellular repulsive forces remain unaffected. Liao Fulong et al. investigated the effect of red peony extract on RBCs aggregation and reported a significant inhibitory effect, which may be mechanistically related to improved RBCs membrane fluidity and reduced fibrinogen levels (Houston et al., 2010).

This study is the first to report that *Bacillus velezensis* is capable of producing proteinaceous metabolites with anticoagulant activity. *In vitro* anticoagulant assays revealed that the anticoagulant protein inhibits fibrin clot formation in a concentration-dependent manner. Notably, Velefibrinase, produced by *Bacillus velezensis* Z01, primarily exerts its effect by degrading preformed fibrin (i.e., thrombolytic activity) (Zhou et al., 2022), whereas the anticoagulant protein identified in this study mainly inhibits fibrin formation (i.e., anticoagulant activity). These diverse mechanisms suggest that different strains of the same species may produce multiple anticoagulant substances targeting distinct steps of the coagulation cascade. This study provides the first evidence of proteins/peptides with fibrin formation-inhibitory activity in this strain, which not only expands the functional spectrum of *Bacillus velezensis* metabolites but also highlights that the potential biomedical applications of this strain remain to be further explored.

Intravascular thrombosis plays a critical pathogenic role in cardiovascular diseases. Under the catalysis of coagulation factor XIIIa, the αA, βB, and γ peptide chains of fibrinogen undergo cross-linking via disulfide bonds, leading to blood clot formation. Therefore, investigating the degradation of fibrinogen by anticoagulant proteins is essential for elucidating their functions in thrombotic or anticoagulant processes (Singh et al., 2023). SDS-PAGE reduction analysis revealed that the anticoagulant proteins primarily cleaved the γ chain of fibrinogen, followed by the Bβ chain, whereas the Aα chain remained largely intact after 180 min of incubation, with only minor degradation observed. This degradation order (γ > Bβ > Aα) is distinctly different from that of classical fibrinolytic enzymes, which preferentially degrade the α chain. Previous studies have reported that microbial proteases typically degrade fibrinogen in the order Aα > Bβ > γ (Jiang et al., 2024). However, some research has indicated that the fibrinogen-binding protein from *Bacteroides fragilis* degrades fibrinogen by first targeting the Bβ and γ chains, followed by the Aα chain (Houston et al., 2010). Jin Huang et al. reported that a novel protein isolated from the venom of *Agkistrodon acutus* exerts anticoagulant effects by cleaving the Aα, Bβ, and γ chains of fibrinogen (Huang et al., 2021). Nevertheless, the degradation sequence observed in this study (γ > Bβ > Aα) remains a rarely reported phenomenon.

This atypical degradation pattern is characterized by the susceptibility of the γ chain to degradation, whereas the Aα chain exhibits resistance. The underlying mechanism may involve the following two factors. First, the Aα chain can undergo conformational changes through post-translational modifications (e.g., acetylation and phosphorylation) (Nencini et al., 2024), thereby resisting proteolytic hydrolysis. Second, the C-terminal aggregation domain of the γ chain, which contains a calcium-binding site and a deep binding pocket that provides an aggregation surface, represents the most variable region within the fibrinogen-related protein family (Yee et al., 1997). This domain is rich in acidic residues and displays high structural flexibility, making it prone to recognition and subsequent degradation by anticoagulant proteins.

Furthermore, the polymerization domain of the γ chain plays a guiding role in fibrin monomer assembly, and its structural integrity is essential for proper fibrin formation (Yee et al., 1997). Depletion of the γ chain disrupts fibrin formation, thereby exerting an anticoagulant effect. Therefore, in the present study, even when the Aα chain remained largely intact, degradation of the γ chain still impeded fibrin polymerization, potentially contributing to an anticoagulant effect. This observation is consistent with the finding that the anticoagulant protein characterized herein inhibits fibrin polymerization and exhibits anticoagulant activity (as described above, 3.6.). Notably, such substrate specificity has rarely been reported among microbial proteases. Thus, this anticoagulant protein is a promising novel anticoagulant candidate.

Fibrinogen Bβ chain also plays an essential role in fibrin polymerization (Mosesson, 1997; Moen et al., 2003). Moreover, its fragment, Bβ15-42, has been shown to inhibit both platelet aggregation and fibrinogen binding (Chen et al., 1988). Consequently, degradation of the Bβ chain may confer dual effects on the anticoagulant protein, manifesting as both anticoagulant and antiplatelet activities.

Unlike traditional anticoagulants such as heparin, warfarin, and direct oral anticoagulants (DOACs), which act by globally suppressing coagulation factor activity or synthesis, the anticoagulant protein characterized in this study targets fibrinogen directly. This mechanism-based distinction is significant because conventional agents, despite their established efficacy, are invariably associated with a persistent risk of bleeding complications (Palareti, 2014; Kholmukhamedov et al., 2025). The direct degradation of fibrinogen may circumvent the broad coagulopathy induced by factor inhibition, offering a more circumscribed anticoagulant effect. This is consistent with the emerging paradigm in anticoagulant development that seeks to decouple antithrombotic efficacy from bleeding risk through more precisely targeted interventions (Nasiri et al., 2025).

Despite the encouraging *in vitro* safety results described above, it should be noted that hemolysis assays cannot fully replicate the complex *in vivo* environment (e.g., blood flow shear stress, plasma protein binding, immune interactions). Additionally, the present study focused on erythrocyte toxicity and did not examine effects on other blood cells or vascular endothelial cells. Nevertheless, these limitations do not diminish the main conclusions regarding the anticoagulant activity and hemocompatibility of the anticoagulant protein within the tested *in vitro* conditions. Future studies using animal models are warranted to evaluate systemic safety and bleeding risk.

## 5 Conclusion

An anticoagulant protein was obtained from the metabolites of *Bacillus velezensis* B01, a strain isolated from mangrove sediment. The protein has a molecular weight of 27.2 kDa, exhibits no hemolytic activity, and inhibits both erythrocyte aggregation and fibrin formation. It degrades fibrinogen in a substrate-specific manner, with a degradation order of γ > Bβ > Aα, a pattern that indicates novelty. Collectively, this anticoagulant protein shows potential as a candidate for clinical anticoagulant therapy.

## Data Availability

The original contributions presented in the study are included in the article/supplementary material, further inquiries can be directed to the corresponding author.
